# Resident mucosal programmes in colon cancer: organ-associated formation and remodelling of the immune niche

**DOI:** 10.3389/fimmu.2026.1980704

**Published:** 2026-10-29

**Authors:** Wenjun Jiang, Bochen Pan, Zijuan Song, Zhongcheng Zhang, Haifan Yan

**Affiliations:** 1 Department of Digestive Diseases, The First People's Hospital of Wenling (Taizhou University Affiliated Wenling Hospital), School of Medicine, Taizhou University, Wenling, Zhejiang, China; 2 Tongji University Cancer Center, Shanghai Tenth People's Hospital, School of Medicine, Tongji University, Shanghai, China

**Keywords:** colon cancer, immune remodelling, mucosal immunity, organ-associated immunity, tumour co-option, tumour immune niche

## Abstract

Resident epithelial, immune and stromal cells organise immune responses in the colonic mucosa, but their contribution to the immune environment of colon cancer remains incompletely understood. We propose that colonic tumours may partly use pre-existing mucosal cells and regulatory mechanisms, a possibility we refer to as co-option. Chronic colitis provides a comparison for examining changes in mucosal immunity, without implying that inflammation is a necessary precursor to sporadic colon cancer. Colonic BTNL3/BTNL8 proteins regulate resident γδ T-cell subsets, and compartmentalised antigen presentation shapes local immune responses. In mouse models of colitis-associated cancer, resident stromal cells give rise to MCAM^+^ cancer-associated fibroblasts that promote macrophage recruitment through IL-34/CCL8 signalling. Tumour-associated immune changes include shifts in γδ T-cell subset composition and altered functions of RORγt^+^ regulatory T cells. MHC class II-dependent interactions between group 3 innate lymphoid cells and T cells also support microbiota-dependent antitumour immunity. Shared signalling is exemplified by the IL-22–oncostatin M–STAT3 pathway in experimental colitis and colitis-associated cancer. However, similarity in cell states and signalling does not establish that tumours preserve pre-existing multicellular mucosal niches. Much of the causal evidence derives from colitis-associated cancer models, and its relevance to sporadic colon cancer remains uncertain. Whether tumours use the same mucosal cells and regulatory mechanisms requires direct testing. Defining tumour-associated changes in these mechanisms may reveal therapeutic targets while preserving protective intestinal immunity.

## 1 Introduction

Immune checkpoint inhibition has redrawn treatment algorithms across solid tumours, yet colorectal cancer (CRC) remains among the most uneven beneficiaries (1). Durable responses are largely confined to the mismatch repair-deficient (dMMR)/microsatellite instability-high (MSI-H) subset (2), which accounts for only about 5% of metastatic disease (3), a major setting in which these agents are approved (4–6). Tumour-intrinsic features, particularly mutational burden, antigenicity and associated immune activation, explain an important part of this heterogeneity (7) but do not fully account for the organisation of the local immune landscape. This raises the question of whether pre-existing mucosal mechanisms influence checkpoint-blockade responses through antigen presentation, immune-cell positioning and effector function.

An additional level of explanation lies in the tissue context. The intestinal mucosa actively organises persistent, spatially and functionally structured immune responses. In inflammatory bowel disease (IBD), it can assemble stable multicellular inflammatory programmes, including a pathogenic cellular module in ileal lesions that predicts resistance to anti-tumour necrosis factor therapy (8, 9). Such responses are not equivalent to antitumour immunity, but they demonstrate the capacity of intestinal tissue to organise and maintain complex local immune states. Inflamed colonic mucosa also carries excess carcinoma risk (10). Importantly, chronic inflammation is relevant here because it reveals the plasticity of resident mucosal immune organisation, rather than because it is assumed to represent an obligatory precursor of sporadic CRC. CRC immune quiescence may therefore reflect active local regulation rather than an intrinsic inability of colonic tissue to organise immunity.

Two adjacent literatures address different aspects of this problem. Clinically oriented syntheses of IBD-associated CRC centre on cancer risk, surveillance and management (11), while mechanistic accounts of early immune evasion emphasise malignant epithelial states, stem-cell dynamics and driver mutations (12). These perspectives provide important tumour-centred explanations but do not systematically separate tumour-associated immune organisation from the contribution of pre-existing colonic mucosal programmes. The unresolved question is therefore how much of the immune organisation observed in colonic tumours is imposed *de novo* by malignancy and how much reflects the changes in resident mucosal cells and their regulatory mechanisms.

We therefore examine whether colonic tumours use cells and immune-regulating mechanisms already present in non-malignant colonic mucosa, a possibility we refer to as co-option. We focus on epithelial regulation of resident lymphocytes, changes in immune-cell composition and stromal-cell function during tumour development, and signalling pathways shared by colitis and cancer. Inflamed non-malignant mucosa provides a comparison for examining how these cells and mechanisms change, without implying that colitis is a necessary precursor to sporadic colon cancer. Comparisons with normal colon, as well as with non-colonic tumours and their corresponding non-malignant tissues, could help distinguish features associated with colonic mucosa from immune changes common to different cancers. However, similar cell states or signalling pathways alone do not establish co-option. Additional evidence must link pre-existing mucosal cells or mechanisms to their functional use within the tumour immune environment.

## 2 Defining an organ-associated immune niche: evidence and attribution

### 2.1 Evidence for an organised immune niche

Here, an immune niche denotes a local, spatially organised assemblage of immune cells and tissue partners linked by functional interactions. We evaluate evidence for such niches across four complementary dimensions: cellular composition, spatial organisation, sustaining mechanisms and temporal dynamics.

Cellular composition identifies the populations and states involved. Single-cell profiling of paired colorectal tumours and liver metastases has resolved diverse lymphoid and myeloid populations, including exhausted CD8^+^ T cells, activated regulatory T cells, NK cells, mucosal-associated invariant T cells, and distinct dendritic-cell and macrophage subsets (13). Their presence alone, however, does not establish an organised niche.

Spatial organisation addresses how these populations are arranged. Multiplexed imaging identified nine recurring cellular neighbourhoods at the invasive front of advanced CRC, with differences in their organisation associated with clinical outcomes (14). Tumour–stroma boundary organisation has also been associated with checkpoint blockade response (15).

Functional perturbation tests regulatory dependence. In colitis-associated cancer models, epithelial IFNγ signalling and compartmentalised antigen presentation regulate inflammatory responses and tumour burden through pathways involving extracellular ATP, macrophage NLRP3 activation and IL-1 signalling (16). These findings provide causal evidence for a multicellular regulatory circuit, although they do not directly establish how a spatially organised niche is maintained.

Stage-associated variation provides indirect evidence of remodelling. In human CRC, later tumour stages were associated with higher densities of T follicular helper and innate immune cells, but lower densities of most T-cell populations (17). These cross-sectional findings do not establish how individual niches change over time.

Evidence for a particular niche is strongest when these complementary approaches converge on the same cellular arrangement and its function.

### 2.2 Attributing niche features to the organ and the tumour

An organised niche may be shaped by the organ, the tumour or both. Attribution is therefore distinct from niche identification.

Analysis of paired colorectal tumours, liver metastases and non-malignant tissues compared immune-cell phenotypes across tissues to distinguish malignancy-associated, niche-associated and combination-associated immune states (13). We draw on this comparative logic without transferring its classifications directly to primary colonic tumours.

For BTNL-responsive γδ T cells, RORγt^+^ regulatory T cells and ILC3s, the relevant question is whether tumour-associated populations retain or modify cellular programmes and regulatory dependencies found in non-malignant colonic mucosa. Comparisons with non-colonic tumours and their corresponding non-malignant tissues could help distinguish organ-associated features from broadly shared tumour-associated states. Organ association does not require a population to be absent from other tissues.

Such similarities would be consistent with a mucosal contribution, but establishing co-option requires additional evidence linking pre-existing mucosal cells or mechanisms to their functional use in tumours.

## 3 The colon as an organ-associated immune-organising environment

The colonic epithelium regulates resident immune cells through BTNL signalling and antigen presentation. Interactions between the microbiota, ILC3s and regulatory T cells also contribute to mucosal immune regulation. These mechanisms may influence the tumour immune environment, but whether tumours use the same pre-existing mucosal mechanisms requires direct testing.

### 3.1 Epithelial organisation of resident immunity

Epithelial butyrophilin-like molecules provide one of the clearest examples of epithelial regulation of resident intestinal γδ T cells. Murine Btnl1 shapes and maintains the intestinal Vγ7^+^ compartment, while human colonic BTNL3/BTNL8 selectively regulate colonic Vγ4^+^ cells (18, 19). Importantly, continued epithelial Btnl expression is required to preserve the characteristic Vγ7^+^ phenotype, and distinct BTNL heteromer combinations support different intestinal γδ T-cell repertoires (19, 20). This places BTNL signalling not only upstream of initial compartment specification but also in the ongoing maintenance of resident immune identity. Colonic CD103^+^Vγ4^+^ cells are reduced in inflammatory bowel disease, and BTNL3/BTNL8 hypomorphism is associated with penetrating Crohn’s disease (21). Small-intestinal coeliac disease further shows that chronic inflammation can durably remodel a BTNL-associated resident compartment even after epithelial BTNL8 expression recovers (22). These findings establish a role for epithelial BTNL signalling in shaping and maintaining resident intestinal γδ T-cell populations, although direct dependence of tumour-associated γδ T cells on this mechanism has not been demonstrated.

Epithelial organisation also extends to antigen presentation (23). In murine colitis, epithelial IFNγ signalling promotes MHC-I and MHC-II antigen presentation to CD8αβ^+^ and CD4^+^ intraepithelial T cells, respectively, inducing CD39 (ENTPD1) and ENTPD5 expression. This limits extracellular ATP accumulation, restraining macrophage NLRP3 activation and IL-1 production. Conversely, macrophage MHC-II presentation and IL-1 support pathogenic GM-CSF-producing CD4^+^ T cells. In the azoxymethane–dextran sulfate sodium (AOM–DSS) model of colitis-associated cancer, epithelial deletion of Ifngr1 (encoding IFNγR1) impairs this signalling, whereas B2m deletion disrupts MHC-I presentation; both reduce T-cell CD39 expression and increase tumour burden. Apyrase, MCC950-mediated NLRP3 inhibition or IL-1R blockade reduces tumour burden in epithelial Ifngr1-deficient mice (16). This murine evidence does not establish co-option or applicability to sporadic human CRC.

### 3.2 Microbiota-responsive mucosal programmes

Fusobacterium nucleatum is enriched in CRC (24, 25), and its Fap2 protein inhibits NK- and T-cell activity through TIGIT (26). These observations establish microbial enrichment and direct immune suppression, but do not demonstrate co-option of a pre-existing mucosal programme. Similarly, deoxycholic acid suppresses CD8^+^ T-cell effector function (27), but this does not establish colon specificity or reuse of a resident immune circuit.

Mucosal immune regulation provides a separate line of evidence. In mice, MHC class II-dependent ILC3–T-cell interactions support microbial communities that promote type 1 immunity in the intestine and tumours; loss of ILC3-specific MHC class II promotes invasive cancer and resistance to PD-1 blockade (28). Commensals induce colonic Tregs, including RORγt^+^ subsets (29, 30), while microbial metabolites promote peripheral Treg generation (31). ILC3-mediated antigen presentation also supports microbiota-specific RORγt^+^ Treg selection (32). Human colon cancers contain Foxp3^+^RORγt^+^ Tregs with potent T-cell-suppressive but compromised anti-inflammatory activity (33), and related populations sustain tumour growth in murine colitis-associated cancer (34).

### 3.3 Additional mucosal immune populations

Colonic CD8^+^ tissue-resident memory T cells show clonal expansion and altered inflammatory programmes in ulcerative colitis (35). In human CRC, PD-1^+^ICOS^+^ CD4^+^ T cells display a resident-memory phenotype and local proliferation (36). Tumour-infiltrating mucosal-associated invariant T (MAIT) cells show evidence of bacterial-antigen recognition and an exhausted CD39^+^ phenotype (37). In murine colitis-associated cancer, Notch2 signalling in conventional dendritic cells supports their differentiation, migration and antigen cross-presentation to CD8^+^ T cells, restraining tumourigenesis (38). These findings do not establish that tumour-associated populations persist from pre-existing mucosal cells; resident-memory phenotypes and local immune functions alone are insufficient evidence of co-option.

These studies identify mucosal regulatory mechanisms and tumour-associated immune states. Whether tumour populations persist from resident compartments, are newly recruited or acquire similar states independently remains unresolved. Demonstrating co-option requires evidence linking pre-existing mucosal cells or mechanisms to their functional use in tumours.

## 4 Building and remodelling the immune niche through resident mucosal components

Two examples illustrate potential contributions of resident colonic populations: expansion and functional alteration of stromal cells, and changes in γδ T-cell subset composition (**Figure 1**).

**Figure 1:**
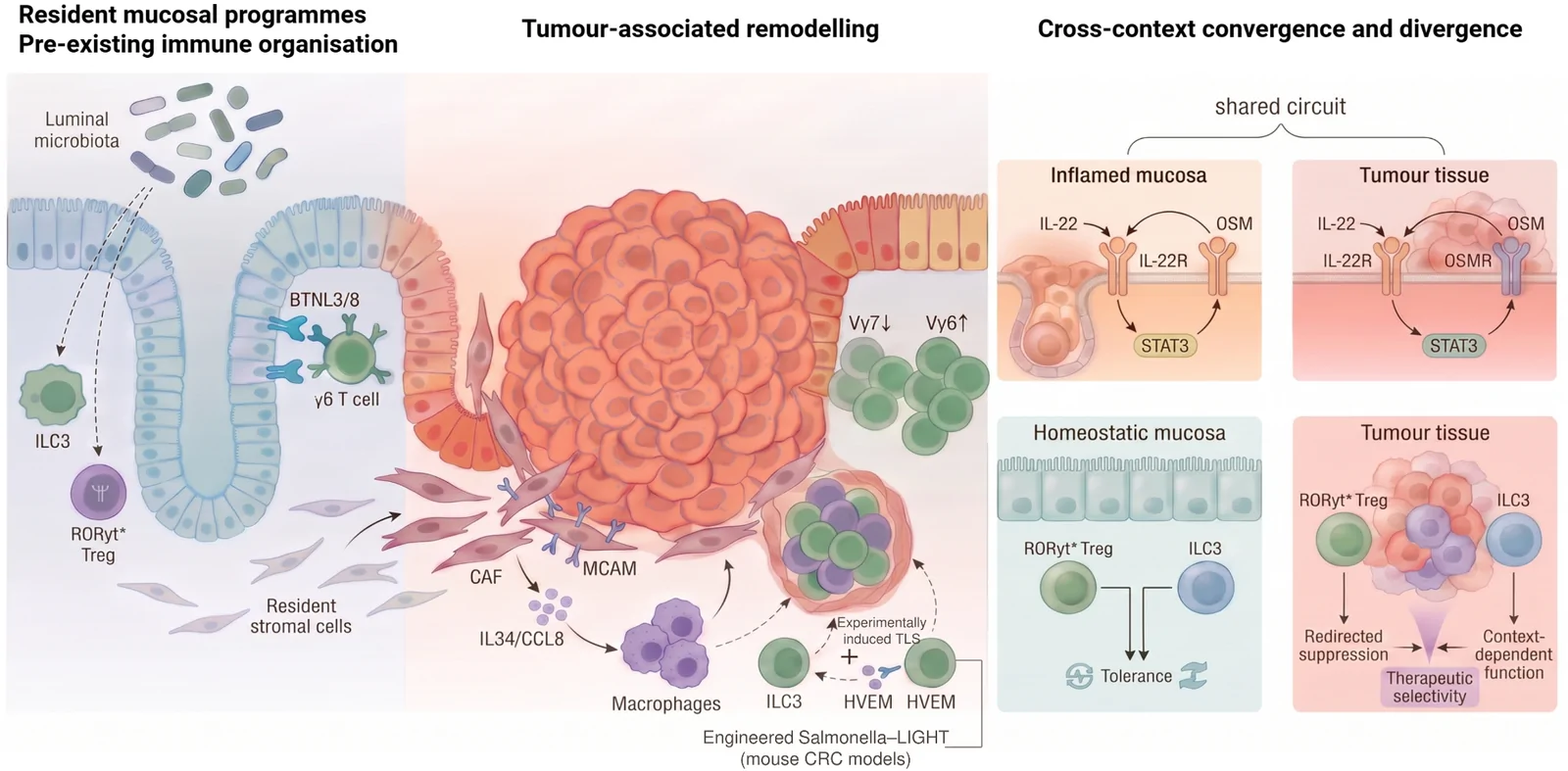
Proposed remodelling of resident mucosal programmes in the colonic tumour immune niche. The schematic summarises how epithelial signals, resident immune cells and stromal cells in the colonic mucosa may contribute to the tumour immune environment. The mucosal components shown include epithelial BTNL3/BTNL8 signalling, microbiota-responsive ILC3s and RORγt^+^ Tregs, and resident stromal populations. Tumour-associated changes include MCAM^+^ fibroblast-associated macrophage recruitment and shifts in γδ T-cell subset composition. The LIGHT–HVEM–ILC3/TLS branch separately depicts experimental induction by LIGHT-expressing Salmonella in mouse CRC models. It illustrates host responsiveness to an exogenous intervention, without establishing spontaneous tumour co-option or derivation of tumour TLSs from pre-existing mucosal lymphoid structures. Cross-context comparison highlights circuit-level convergence, exemplified by IL-22–OSM–STAT3 signalling, alongside context-dependent functional divergence of resident immune programmes. The model is conceptual and does not imply direct lineage continuity or a required progression from chronic inflammation to sporadic colorectal cancer.

### 4.1 Expansion and functional conversion of resident stromal cells

A stromal component of the tumour niche can arise from cells already resident in the normal colon. Lineage tracing in a colitis-associated cancer model shows that many ACTA2^+^ cancer-associated fibroblasts derive from intestinal pericryptal leptin receptor-positive cells rather than recruited precursors (39). Their progeny acquire MCAM, whose expression is induced by transforming growth factor β and localises to colorectal tumour stroma. Functionally, stromal Mcam deletion reduces tumour-associated macrophage recruitment and tumour growth, with fibroblast MCAM engaging IL1R1 to augment NF-κB-dependent IL34/CCL8 signalling (39). These findings show that tumour-associated immune organisation can arise partly through expansion and functional conversion of an organ-resident stromal population. The lineage evidence is murine and inflammation-associated, whereas the human evidence is limited to stromal expression and outcome associations (39).

### 4.2 Changes in γδ T-cell subset composition during tumour development

The resident γδ T-cell compartment is also remodelled during tumour development. Premalignant or nontumour colon is enriched in cytotoxic γδ T-cell states, whereas tumour-infiltrating populations show protumorigenic features associated with distinct TCR-Vγδ usage in humans and mice (40). In colitis-associated cancer, Vγ6^+^ cells increase as Vγ7^+^ cells decrease, and functional targeting of these subsets produces opposing effects on tumour growth (40). The pattern therefore supports changes in the relative abundance of distinct γδ T-cell subsets rather than linear conversion of one population into another. It is consistent with remodelling of an organ-structured immune compartment, but does not establish direct developmental continuity in human tumours.

Lineage tracing supports stromal origins in murine colitis-associated cancer, whereas γδ T-cell studies demonstrate altered subset composition and function. Neither establishes inheritance of a complete mucosal immune niche.

## 5 Shared inflammatory circuits and context-specific divergence

Current evidence is stronger for convergence between inflamed mucosa and tumour at the level of signalling circuits than for preservation of complete multicellular modules. Interventional evidence can establish shared pathway dependence, whereas resemblance in cellular states or modules remains descriptive unless supported by spatial, functional or clonal continuity (**Table 1**). Cross-context similarity may also reflect generic inflammatory responses rather than organ-associated co-option.

**Table 1:** Convergent and divergent immune features across inflammatory bowel disease, colitis-associated cancer and sporadic colorectal cancer.

Feature	IBD (UC/CD)	Colitis-associated cancer	Sporadic CRC	Cross-context interpretation	Evidence strength	Key refs
Epithelial BTNL–γδ T-cell selection	CD103^+^Vγ4^+^ cells decrease in active IBD; BTNL3/8 hypomorphism is associated with penetrating CD	—	—	Cancer relevance remains untested	IBD: strong; cancer: insufficient	(18, 19, 21, 22, 41)
Compartmentalised antigen presentation	No direct comparable human evidence	Epithelial IFNγR loss increases tumour burden in AOM–DSS CAC	—	Shared inflammatory mechanism; sporadic relevance untested	CAC: strong; sporadic CRC: insufficient	(16)
ILC3 abundance and MHC-II-dependent regulation	Reduced ILC3 frequencies in UC mucosa	ILC3-specific MHC-II deletion increases tumour burden and invasion in AOM–DSS mice	Reduced ILC3 frequencies and altered ILC3 states in human tumours	Shared cell population; continuity and co-option unproven	Human: emerging; CAC models: strong; co-option: insufficient	(28, 42)
IL-22–OSM epithelial circuit	IL-22 induces OSMR; OSMR correlates with IL22 enrichment in UC	Epithelial OSMR deletion attenuates colitis/CAC; OSM blockade reduces tumour growth	—	Convergent in colitis and CAC; sporadic relevance untested	CAC: strong; sporadic CRC: insufficient	(43, 44)
γδ T-cell phenotype	No direct matched comparison	Vγ7^+^ cells decrease and Vγ6^+^ cells increase in tumour regions	Tumour-infiltrating γδ T cells shift toward protumour subsets with distinct TCR usage	Shared compartment, divergent subset usage	Emerging	(40)
MAIT cells	Reduced and pro-apoptotic; pathogenic activity supported in murine colitis	—	Tumour-infiltrating MAIT cells show bacterial-antigen recognition, exhaustion and CRC-restricted CD39^+^ states	Cross-disease relationship remains unresolved	IBD: emerging; CRC: emerging	(37, 45, 46)
IgA/plasma cells	Altered secretory IgA biology	IL-10^+^ B-cell-derived IgA plasma cells restrain CAC	IgA plasma-cell dysfunction is associated with poor outcome in *Fusobacterium*-infected CRC	Functional divergence across contexts	Emerging	(47–49)
Inflammatory fibroblasts and SPP1^+^ macrophages	SPP1^+^ macrophages and CHI3L1^+^ fibroblasts are enriched in UC	Resident Lepr^+^ stromal cells generate ACTA2^+^/MCAM^+^ CAFs	Stromal MCAM expression is associated with poorer survival	Convergent stromal remodelling	UC: emerging; lineage evidence: strong	(39, 50–52)
Neutrophils/NETs	NETs sustain inflammatory signalling; butyrate restrains neutrophil activity	—	Tumour-associated neutrophil states emerge; *F. nucleatum* promotes NET-dependent progression	Cross-disease relationship remains unresolved	IBD: emerging; CRC: emerging	(53–56)
Treg programme and gut homing	Microbiota-shaped RORγt^+^ Tregs and disease-specific homing programmes	RORγt^+^ Tregs promote CAC through dendritic-cell IL-6	RORγt^+^Foxp3^+^ Tregs retain suppressive activity but show impaired anti-inflammatory function	Shared compartment, divergent function	Strong	(29–34, 57–63)
Microbiota and metabolites	Butyrate promotes peripheral Treg generation	—	*Fusobacterium*–Fap2–TIGIT signalling suppresses cytotoxic immunity; deoxycholic acid impairs CD8^+^ effector function	Shared microbial instruction, divergent effects	Mechanism: strong; colon specificity: insufficient	(24–27, 31)

UC, ulcerative colitis; CD, Crohn’s disease; CAC, colitis-associated cancer; CRC, colorectal cancer; TLS, tertiary lymphoid structures; NETs, neutrophil extracellular traps. Strong indicates direct perturbational or functional evidence within the specified experimental or clinical context; emerging, observational or partial causal evidence; and insufficient, no direct evidence for the stated comparison. A dash (—) denotes an evidence gap rather than evidence of absence.

### 5.1 Circuit-level convergence

The IL-22–oncostatin M (OSM) axis provides one of the clearest examples of circuit-level convergence between intestinal inflammation and tumourigenesis. IL-22 induces epithelial OSMR through STAT3, allowing OSM to reinforce STAT3 activation, inflammatory adaptation and immune-cell chemotaxis (43). Conditional epithelial OSMR deletion protects mice from both colitis and colitis-associated cancer, while pharmacological OSM blockade attenuates established tumours (43). Human colonic organoids and ulcerative colitis cohorts further support activation of this circuit in inflammatory mucosa (43), whereas IBD-associated colorectal cancers show OSMR dysregulation (44). Thus, shared IL-22–OSM signalling is well supported in inflammation-associated carcinogenesis, but its use in sporadic colon cancer remains unresolved. More generally, models that experimentally couple inflammation and carcinogenesis cannot independently establish the same circuitry in sporadic disease.

### 5.2 Multicellular continuity remains uncertain

Evidence for continuity of complete multicellular modules is weaker. Single-cell analysis of ileal Crohn’s disease identified a pathogenic module comprising plasma cells, inflammatory mononuclear phagocytes, activated T cells and stromal cells associated with anti-TNF resistance (8). Related inflammatory states are also seen in colonic mucosa, including SPP1^+^ macrophages and CHI3L1^+^ fibroblasts in ulcerative colitis (51, 64). However, recurrence of similar cell states across inflammatory and tumour contexts does not establish preservation of the same multicellular niche architecture.

### 5.3 Functional divergence

Functional divergence may be more therapeutically informative than similarity itself. Resident mucosal populations can be reprogrammed during inflammation: checkpoint inhibitor colitis and ulcerative colitis both involve activation of tissue-resident T-cell states (35, 65–67). These observations show that resident identity does not imply functional stability when the surrounding inflammatory context changes. Human colon cancer Foxp3^+^RORγt^+^ Tregs retain T-cell-suppressive activity but show impaired anti-inflammatory function (33), whereas phenotypically related RORγt^+^ Tregs in murine colitis-associated cancer can promote tumour growth by permitting dendritic-cell IL-6 activity (34). These findings highlight context-dependent immune functions, without establishing continuity between pre-existing mucosal cells and tumour populations. Defining tumour-associated functional changes may therefore help identify therapeutic targets while preserving protective intestinal immunity.

## 6 Therapeutic implications, evidence gaps and decisive tests

Therapeutic implications depend on whether the relevant feature is a shared mucosal circuit, a tumour immune phenotype or a programme required for intestinal homeostasis. The IL-22–OSM axis illustrates the first case: OSMR targeting is clinically tractable in inflammatory disease, but current tumour evidence remains predominantly murine and inflammation-associated (43). Its relevance therefore lies in demonstrating pharmacological accessibility of a shared mucosal circuit rather than clinical validation in colon cancer. Conversely, interference with programmes shared by normal mucosa may carry a cost to tissue immunity, as illustrated by gut-selective α4β7 blockade in colitis (68, 69).

Experimental induction of tertiary lymphoid structures (TLSs) offers a potential therapeutic strategy. In mouse CRC models, engineered Salmonella delivers LIGHT and promotes mature TLS formation; HVEM or ILC3 deficiency reduces this response (70). This demonstrates an experimentally inducible host response, but does not establish the same initiating pathway in spontaneous human CRC or derivation of tumour TLSs from pre-existing mucosal lymphoid structures.

Local immune organisation may help explain why antigenic potential does not uniformly translate into checkpoint-blockade benefit. Tumour–stroma boundary organisation associates with clinical response (15), and CD74-marked interferon-high states associate with benefit from ICI-containing treatment (71). In mice, loss of ILC3-specific MHC class II disrupts microbiota-dependent type 1 immunity and promotes resistance to PD-1 blockade (28). These findings link local immune organisation to treatment response, but do not establish that the relevant tumour states arise through co-option of pre-existing mucosal programmes. Whether features reflecting such co-option improve response prediction beyond MMR/MSI status and tumour mutational burden requires direct validation.

Co-option should therefore be treated as a testable framework rather than an established lineage history. A decisive comparison would include normal colon, inflamed non-malignant mucosa, sporadic colon cancer, colitis-associated cancer and non-colonic primary tumours, using paired spatial, clonal and perturbational analyses where possible (72). Alignment of colonic tumour immune states with cross-organ malignancy-associated programmes would favour tumour-driven reprogramming, whereas reproducible phenotypic, spatial or clonal continuity with colonic mucosal states and dependence on shared organising signals would strengthen an organ-associated co-option model. Until such evidence is available, tumour-specific functional divergence may offer a more selective therapeutic direction than indiscriminate targeting of mucosal programmes required for intestinal homeostasis.
